# The histopathological effects of sleep disorders on striated muscle in rats

**DOI:** 10.15537/smj.2023.44.4.20220714

**Published:** 2023-04

**Authors:** İlkay Çinar, Muhammed Bozoğlan, Kürşad Aytekin, Deniz Esenyel, Cem Zeki Esenyel

**Affiliations:** *From the Department of Pathology (Çinar) and Department of Orthopaedic Surgery (Aytekin, Esenyel C), Faculty of Medicine, Giresun University, Giresun; From the Department of Orthopaedic Surgery (Bozoğlan), İzmir Health Sciences University Tepecik Training and Research Hospital, İzmir; Department of Plastic and Reconstructive Surgery (Esenyel D), Kartal Dr. Lütfi Kırdar Training and Research Hospital, İstanbul, Turkey.*

**Keywords:** sleep disorder, muscle, degeneration, atrophy, immunohistochemistry, IGF1

## Abstract

**Objectives:**

To histopathologically examine the change in gastrocnemius muscle created by sleep disorder in rats.

**Methods:**

This study was carried out at Giresun University, Turkey from December 2018 to January 2021. A total of 30 Wistar rats were separated into 3 groups as the control group (CG), absence of rapid eye movement (REM) sleep (ARS) group, chronic absence of sleep (CAS) group. The lack of sleep was created in all rats. At the end of 21 days, all the rats were euthanized. Degeneration and regeneration findings, and expressions of muscle RING finger 1 (MuRF1), muscle atrophy F-box (MAFbx), tumor necrosis factor (TNF), cyclooxygenase 2 (COX 2), insulin-like growth factor 1 (IGF1) in the gastrocnemius muscles were evaluated histopathologically and immunohistochemically.

**Results:**

Degeneration was found to be greater in the ARS and CAS groups compared to the CG. Regeneration was determined to be significantly lower in the CAS group compared to the ARS group and control group. The number of atrophic fibres was greater in the CAS and ARS groups than in the control group. The IGF1 staining in the CAS group was found to be stronger than in the other 2 groups.

**Conclusion:**

This study demonstrated an increase in findings of degeneration in the gastrocnemius muscle of rats with a lack of sleep. The regeneration was reduced in the group with chronic lack of sleep.

**S**leep is one of the most important requirements for a healthy life, and is an active regeneration period, which prepares the whole body for life again.^1-3^ This unconscious state, which is experienced regularly at certain hours of the day appropriate to a circadian rhythm, restores the body for healthy preparation for the next day. Periods of rapid eye moment (REM) and non-REM (NREM) sleep follow each other periodically during sleep.^4^

Sleep disorders include problems related to the amount and quality of sleep (such as insomnia, over-sleeping, rhythm changes) or abnormal events experienced during sleep (such as somnambulism, nightmares, bruxism, snoring). In addition to anxiety and psychiatric problems, there is a substantial amount of data that lack of sleep also causes dysfunction in several systems including the muscles and skeletal system. A high prevalence of complaints of chronic pain in the musculoskeletal system has been determined in individuals with sleep disorders.^4,5^ At least 90% of patients who present at hospital because of pain have a sleep problem.^4^ There are many studies in literature which have shown a relationship between sleep disorders and muscle pains, and there are also studies that have reported that lack of sleep leads to reduced muscle mass and fat infiltration.^4-10^ However, there are few experimental studies which have included the histomorphological changes seen in muscles.

In a previous study, it was reported that sleep disorder caused a decrease in the weight and cross-sectional surface area of the tibialis anterior muscle in rats.^6^ In another study, it was shown that muscle regeneration is impaired and insulin-like growth factor 1 (IGF 1) levels is decreased in sleep disorder, and decreased IGF 1 levels can return to basal levels with the improvement of sleep.^9^ However, the number of histomorphological studies on this subject is limited. Investigation of morphological and immunohistochemical changes is necessary to elucidate the effects and mechanism of insomnia on muscle.

The aim of this study was to histopathologically examine the changes in striated muscle tissue in rats with a sleep disorder created, and to objectively reveal the effects of sleep disorder on muscle. To understand the mechanism of the effects of insomnia on muscle, expression of muscle ring finger 1 (MuRF1), Muscle atrophy F-box (MAFbx), tumor necrosis factor (TNF), cyclooxygenase 2 (COX 2), and IGF1 were examined immunohistochemically.

## Methods

This experimental study was carried out at the animal laboratory of Giresun University, Giresun, Turkey, between December 2018 and January 2021. We used the search engine MEDLINE’s electronic databases through PubMed, and Web of Science to search for previous relevant studies. The study was carried out on tissues obtained from animals sacrificed in a study with Ethics Committee approval (decision no:2018/13, dated:18.12.2018). No additional animals were sacrificed for this study. The study approval study was granted by the Ethics Committee (HADYEK: decision no:2019/19, dated:25.12.2019). The work was in accordance with the Animal Research: Reporting of In Vivo Experiments (ARRIVE) guidelines.^11^

A total of 30 male Wistar-albino rats were used in the experiments. Three groups were created: i) control group (n=10); ii) absence of REM sleep group (ARS n=10). iii) chronic absence of sleep group (CAS, n=10). Jouvet’s platform-in-the-water technique was used to create the model of absence of REM sleep and chronic absence of sleep.^12,13^

### Jouvet’s platform-in-the water technique

The control group rats were kept in a wire cage attached above a water tank, with no contact with the water. The rats in the ARS group were placed on metal plates 6.5 cm in width over a water tank. In this way, when the rats fell asleep, they came into contact with the water by sliding on the plate and thus, they could not pass into the REM sleep phase.

The rats in the CAS group were kept on metal plates 14 cm in width, which allowed REM sleep, but contact with the water did not permit deep sleep. All the rats in all 3 groups were kept resting in single cages for one week to become accustomed to the experiment laboratory. In this 1-week period, the rats had free access to food and drinking water in an environment of 24°C room temperature and a 12-hour light-dark cycle (07:00 to 19:00). Following the one-week familiarization, all the groups were accustomed to the experiment apparatus, and to prevent extra falls and reduce stress on the rats, they then underwent a period of familiarization with the devices appropriate to their group for half an hour between 17:00 and 17:30 for 5 days. On the first day after this 5-day period, all the animals were placed in their respective group apparatus at 16:00 and remained there until 10:00 the following morning. At 10:00, they were removed and placed in their resting cages, where they rested until 16:00. The experiment continued in this way for 21 days. To avoid stress on the animals throughout the experiment, they were permitted to socialize within their own group in the experiment cages and in the resting cages. Food and drinking water were provided ad libitum throughout the experiment, and the water tank was cleaned periodically with an automatic system. The day and night system continued in the same way. The general condition of the animals was checked at least twice a day in such a way as not to distract them. At the end of 21 days, all the rats were euthanized with overdose of sodium pentobarbital (Nembutal, Ovation Pharmaceuticals, Deerfield, IL, USA).

### Histopathological and histometric examinations

**Figure 1:**
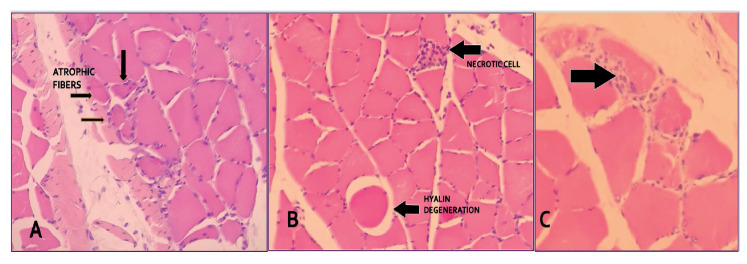
- The histopathological findings of muscle fiber atrophy and degeneration. **A)** Atrophic muscle fibres (chronic absence of sleep group), (hematoxylin and eosin [HE] x20). **B**) Cells showing necrosis and hyalin degeneration within striated muscle tissue, (Absence of REM sleep group), (HE staining x20 magnification). **C**) Lymphocyte infiltration in striated muscle (Chronic absence of sleep group), (HE x20).

**Figure 2:**
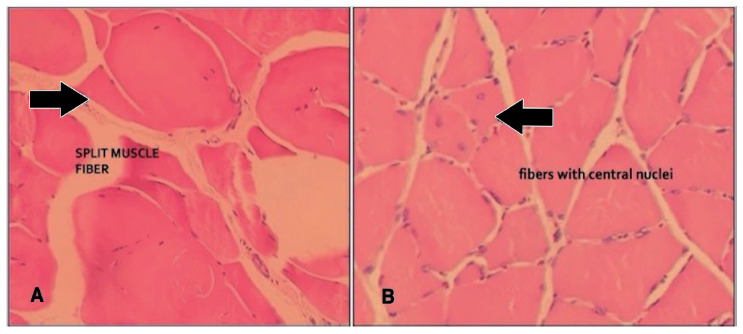
- The histopathological findings of muscle fiber regeneration. **A)** Split striated muscle fibre, (absence of REM sleep group), (HE x40). **B**) Cells with central nucleus in striated muscle, (chronic absence of sleep group), (HE x20).

For the histological and immunohistochemical analyses, the femur-tibia were dissected to include the gastrocnemius muscle and the knee joint. The tissues obtained were fixed in 10% formalin and following routine tissue processing were embedded in paraffin blocks. Preparates were obtained by cutting slices 3 microns in thickness with a microtome from the paraffin blocks. These were then stained with hematoxylin and eosin (HE) and examined under a light microscope. Fat tissue, the presence of connective tissue, and the number of atrophic cells in the tissue were determined in the histomorphological evaluation (Figure 1A). Cells containing signs of degeneration (hyalinized fibre, lymphocytic infiltration, necrosis) were counted (Figures 1B & 1C). Cells containing signs of regeneration (internal nucleus, split fibre, basophilic fibre) were counted (Figures 2). In the histometric and morphological evaluations, 3 different areas at 20 x 100 magnification were evaluated on the HE preparates. Image J 2x program was used in the histometric measurements.

Immunohistochemical staining was examined on a Ventana XT immunohistochemical staining device with routine methods applied in the pathology laboratory. Slices of 3 micron thickness cut with a microtome from the paraffin blocks were stained with anti-MuRF1 antibody (ab201941, Abcam, Cambridge, UK.), anti-Fbx32 antibody (ab157596, Abcam, Cambridge, UK.), anti-COX2 antibody (ab15191, Abcam, Cambridge, UK.), anti-TNF alpha antibody (ab1172, Abcam, Cambridge, UK.), and anti-IGF1 antibody (ab176523, Abcam, Cambridge, UK.). The light microscope evaluations were made by scoring each sample 0, 1, or 2 (0: no staining, 1: mild severity and spread of staining compared to control tissue, 2: strong staining and widespread compared to control tissue).

### Statistical analysis

Power analysis was performed based on previous similar studies, and it was determined that to make a statistical difference it was necessary to have at least 10 subjects in each group. Data obtained in the study were analyzed statistically using the SPSS Statistics for Windows, version 27.0 (IBMCorp, Armonk, NY, USA). Descriptive statistics of the data were stated as mean±standard deviation, median, minimum, and maximum values, number and percentage. Conformity of the data to normal distribution was assessed with the Kolmogorov Smirnov test. In the analysis of independent quantitative data, the ANOVA (Tukey test), Kruskal-Wallis, and Mann Whitney U-tests were used, In the analysis of qualitative data, the Chi-square test was applied, or if conditions were not met, the Fischer test. Each item of data was compared separately between the groups. A total degeneration score was obtained from the number of hyalin fibres, the number of fibres containing lymphocytic infiltration, and the number of necrotic fibres. A total regeneration score was obtained from the number of fibres containing internal nucleus, the number of split fibres, and the number of basophilic fibres. The total scores were compared between the groups. A value of *p*<0.05 was accepted as statistically significant.

## Results

According to the histomorphological evaluation, when the degeneration findings were examined, the number of necrotic fibres was found to be significantly greater in the ARS (*p*=0.0455) and CAS (*p*=0.0432) groups compared to the control group. No statistically significant difference was determined between the ARS and CAS groups (*p*=0.64259). Lymphocyte infiltration was significantly greater in the CAS group than in the control group (*p*=0.0393). No statistically significant difference was determined between the ARS, CAS, and control groups in respect of the number of hyalinized cells. When the number of cells showing findings of degeneration were evaluated in total, the number was statistically significantly higher in the ARS and CAS groups than in the control group (*p*=0.01832 and *p*=0.0268 respectively) (Table 1).

**Table 1:** - Statistical comparisons between the groups of the degeneration findings in the histological evaluation.

Group	Group 2 Necrosis	Group 3 Necrosis	Group 2 Lymphocyte	Group 3 Lymphocyte	Group 2 Degenerated hyalin	Group 3 Degenerated hyalin	Group 2 Total degeneration	Group 3 Total degeneration
Group 3 necrosis	0.6425 (*p*>0.05)							
Group 1 Necrosis	0.0455 (*p*<0.05)	0.0432 (*p*<0.05)						
Group 3 Lymphocyte			0.2408 (*p*>0.05)					
Control Lymphocyte			0.1122 (*p*>0.05)	0.0392 *p*<0.05				
Group 3 Degenerated hyalin					0.2437 (*p*>0.05)			
Group 1 Degenerated hyalin					0.1004 (*p*>0.05)	0.2234 (*p*>0.05)		
Group 3 Total degeneration							0.17264 (*p*>0.05)	
Group 1 Total degeneration							0.01832 *p*<0.05	0.0268 *p*<0.05

Group 1: Control group (n=10), Group 2: Absence of REM sleep group (n=10), Group 3: Chronic absence of sleep group (n=10), Kruskal-wallis (Mann-whitney u test), student t test.

When the regeneration findings were examined, no significant difference was determined between all 3 groups in respect of the number of basophilic cells (*p*=0.473, *p*=0.194, and *p*=0.104) (Table 2). The number of split fibres was determined to be significantly lower in the CAS group compared to the ARS group and the control group (*p*=0.0031 and *p*=0.034) and no significant difference was seen between the ARS and control groups (*p*=0.3768) (Table 2). The number of fibres containing a central nucleus was determined to be significantly lower in the CAS group compared to the ARS group and the control group (*p*=0.03268, and *p*=0.00948) and no significant difference was seen between the ARS and control groups (*p*=0.24632) (Table 2).

**Table 2:** - Statistical comparisons between the groups of the regeneration findings in the histological evaluation.

Groups	Group 2 Regeneratedbasophilia	Group 3 Regeneratedbasophilia	Group 2 Split and ring fibres	Group 3 Split and ring fibres	Group 2 Central nucleus	Group 3 Central nucleus	Group 2 Total regeneration	Group 3 Total regeneration
Group 3 Regenerated basophilia	0.473 (*p*>0.05)							
Group 1 Regenerated basophilia	0.194 (*p*>0.05)	0.104 (*p*>0.05)						
Group 3 Split and ring fibres			0.0031 (*p*<0.05)					
Group 1 Split and ring fibres			0.3768 (*p*>0.05)	0.0347 (*p*<0.05)				
Group 3 Central nucleus					0.03268 (*p*<0.05)			
Group 1 Central nucleus					0.24632 (*p*>0.05)	0.00948 (*p*<0.05)		
Group 3 Total regeneration							0.0433 *p*<0.05	
Group 1 Total regeneration							0.3137 *p*>0.05	0.0221 *p*<0.05

Group 1: control group (n=10), Group 2: absence of REM sleep group (n=10), Group 3: chronic absence of sleep group (n=10), Kruskal-wallis (Mann-whitney u test), and student t test

When the number of fibres showing findings of regeneration were evaluated in total, the number was statistically significantly lower in the CAS group than in the ARS and control groups (*p*=0.0433, and *p*=0.0221) and no significant difference was seen between the ARS and control groups (*p*=0.3137) (Table 2).

In the evaluation of atrophy, there was determined to be a significantly greater number of atrophic fibres in the CAS and ARS groups than in the control group (*p*<0.05) (Kruskal-wallis (Mann-whitney u test). The amount of fat infiltration in the muscle tissue was found to be significantly greater in the CAS group than in the control group (*p*=0.029823). No significant difference was determined between the groups in respect of connective tissue (*p*>0.05) (X² Ki-kare test (Fischer test) (Table 2).

**Figure 3:**
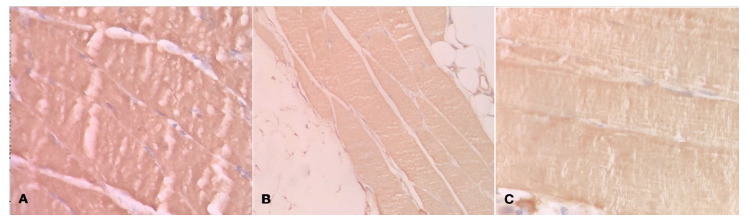
- Immunohistochemical staining of IGF-1 in striated muscle in rats with insomnia. **A**) Control group IGF1 immunochemistry staining (x20). **B)** Absence of REM sleep group IGF1 immunochemistry staining (x20). **C**) Chronic absence of sleep group IGF1 immunochemistry staining (x20).

According to the results of the immunohistochemical examination, the IGF staining in the CAS group was found to be significantly lower than in the ARS and control groups (*p*=0.04957) (Ki-kare test (Fischer test) was used) (Figure 3). No statistically significant difference was determined between the groups in respect of MuRF1, MAFbx, COX2, and TNF expressions (ANOVA and Ki-kare test (Fischer test) were used).

## Discussion

In this study, in which sleep disorder was experimentally created in rats, samples obtained from the gastrocnemius muscle were examined histomorphologically and morphological changes were determined. Tumor necrosis factor-alpha, COX-2, IGF1, MuRF, and MAFbx were examined with immunohistochemical studies.

According to the histopathological analyses, there was an increase in findings of degeneration and a decrease in regeneration in the gastrocnemius muscle of rats where lack of sleep was created. Degenerative changes formed against stress in a muscle group can be seen in the form of necrosis in muscle fibres, granular degeneration, rimmed vacuoles, cytoplasmic bodies, lymphatic infiltration, and hyalinization.^14^ In the current study, the number of necrotic fibres was determined to be significantly greater in both the CAS and ARS groups than in the control group. When necrosis, the number of fibres containing lymphocytic infiltration, and the number of hyalinised fibres were evaluated in total, the total number of cells with findings of degeneration was found to be significantly greater in the 2 groups with sleep disorder than in the control group.

The regeneration of skeletal muscle fibres is histologically related to activated satellite cells. The observation of internal nuclei and cytoplasmic basophilia, and the branching and splitting of muscle fibres is typically associated with regeneration.^15-17^

In the current study, the numbers of split muscle fibres and fibres with central nucleus were significantly lower in the CAS group than in the ARS and control groups. The total number of fibres with findings of regeneration were found to be lower in the CAS group, and no difference was observed between the ARS group and the control group. These findings suggest that regeneration in the muscle is disrupted especially in conditions of chronic lack of sleep.

There is a limited number of studies in literature that have discussed the histomorphological effects of lack of sleep on muscle. In a previous study, it was reported that muscle regeneration was impaired in rats with disrupted REM sleep.^9^ In another experimental rat study, there were determined to be areas of tissue degeneration with intense inflammation in the soleus muscle, and inflammatory infiltrates without tissue degeneration in the plantar muscle.^18^

In the current study, a greater number of atrophic fibres was determined in the 2 groups with sleep disorder. The ratio of fatty tissue was also increased in the CAS group. These results were consistent with the findings of previous studies, which supports the importance of sleep in muscle metabolism, and that lack of sleep can cause muscle atrophy.^6-10^

Muscle ring finger 1 and MAFbx are 2 muscle-specific E3 ubiquitin ligases, which have been shown to be atrophy markers. Muscle ring finger 1 and MAFbx increase in skeletal muscle in conditions that cause atrophy.^19^ In the current study, no significant difference was determined between the groups immunohistochemically in respect of the MuRF and MAFbx values (*p*=0.240). However, observations in previous studies have shown that expression levels of MAFbx or MuRF1 activity can both change or not change.^20^

Insulin-like growth factor 1 stimulates the synthesis of new proteins in skeletal muscles and causes hypertrophy by inhibiting autophagy/apoptosis. The results of the current study showed that IGF expression was lower in the CAS group compared to the other 2 groups (*p*=0.04957). This difference was seen to be correlated with the findings obtained histologically. An increase in degeneration and decrease in regeneration may be related to a decrease in IGF expression in chronic lack of sleep.

Tumor necrosis factor and other inflammatory factors cause protein destruction and inhibition of protein synthesis in muscles.^21^ In the current study, an increase in TNF expression was observed in the gastrocnemius muscles of the rats in which lack of sleep was created. In some previous studies, there has been observed to be an increase in TNF-α in the retroperitoneal fat tissue of rats with lack of sleep.^22^ There has also been reported to be an increase in TNF-α, in other words, in pro-inflammatory expression, in the blood of rats (23). In another study, the TNF-α values in the uvula tissue of individuals with apnea were found to be high (24). In contrast, a study of rats with experimentally created sleep disorder, determined no increase in the TNF-αvalues in the heart, spleen, or liver.^23^ The reason for this difference could have been that some organs are less sensitive to sleep disorders.

Prostaglandin synthesis associated with COX-2 is necessary in the early stages of muscle regeneration. Following traumatic injuries, COX-2 is critical for normal muscle regeneration.^25^ In previous studies, there has been observed to be an increase in the amount of COX-2 in the gastric mucosa of rats with lack of sleep (26). In the current study, the staining of COX-2 was determined to be lower in the 2 groups with sleep disorder compared to the control group, but this decrease was not statistically significant (*p*=0.076). There are also studies showing that COX-2 inhibition is not greatly affected in sleep disorders.^27^

### Study limitations

It is an animal model and may not be match for human muscle. Low sample size was used in our study. Future work are warranted to further confirm our findings using larger sample size.

In conclusion, the results of this study demonstrated that in rats with experimentally created sleep disorders, degeneration developed in the muscles both in the rats lacking REM sleep and in the rats with a chronic lack of sleep. Regeneration did not seem to be affected by a lack of REM sleep, whereas regeneration was reduced in the group with chronic lack of sleep. Insulin-like growth factor 1 is decreased in chronic insomnia.

In conclusion, lack of sleep increased degeneration, had negative effects on regeneration, and could be a cause of atrophy. Our study has concretely demonstrated the degenerative effects of insomnia on muscle. The study also drew attention to the differences between REM and chronic insomnia. The regeneration response was different in REM and chronic disorders. The reduction of IGF1 in chronic insomnia correlates with the regeneration response of the muscle. This degeneration and atrophy could not be statistically associated with MuRF1, MAFbx, COX-2 and TNF expressions, but there is a need for further studies on this subject.
